# Mutational Analysis of Oculocutaneous Albinism: A Compact Review

**DOI:** 10.1155/2014/905472

**Published:** 2014-06-29

**Authors:** Balu Kamaraj, Rituraj Purohit

**Affiliations:** Bioinformatics Division, School of Bio Sciences and Technology (SBST), Vellore Institute of Technology University, Vellore, Tamil Nadu 632014, India

## Abstract

Oculocutaneous albinism (OCA) is an autosomal recessive disorder caused by either complete lack of or a reduction of melanin biosynthesis in the melanocytes. The OCA1A is the most severe type with a complete lack of melanin production throughout life, while the milder forms OCA1B, OCA2, OCA3, and OCA4 show some pigment accumulation over time. Mutations in TYR, OCA2, TYRP1, and SLC45A2 are mainly responsible for causing oculocutaneous albinism. Recently, two new genes SLC24A5 and C10orf11 are identified that are responsible to cause OCA6 and OCA7, respectively. Also a locus has been mapped to the human chromosome 4q24 region which is responsible for genetic cause of OCA5. In this paper, we summarized the clinical and molecular features of OCA genes. Further, we reviewed the screening of pathological mutations of OCA genes and its molecular mechanism of the protein upon mutation by* in silico* approach. We also reviewed TYR (T373K, N371Y, M370T, and P313R), OCA2 (R305W), TYRP1 (R326H and R356Q) mutations and their structural consequences at molecular level. It is observed that the pathological genetic mutations and their structural and functional significance of OCA genes will aid in development of personalized medicine for albinism patients.

## 1. Background

Albinism is a group of disorders caused by reduction of polymeric pigment melanin [1]. Melanin production is closely regulated in the body and occurs in specialized ectodermal derived cells called melanocytes. Generally, melanocytes are possibly cutaneous (hair, skin) or extracutaneous (eye, cochlea) which derived from different ectodermal lineages. The terms albinism, oculocutaneous albinism (OCA), and ocular albinism (OA) can be used both as a phenotypic descriptions and as references to specific syndromes.* Sir Arcibald Garrod* initially states albinism as an inborn error of metabolism [2, 3], but it is now believed to be a heterogeneous genetic disorder caused by mutations in several different genes. Population studies have shown a genetic heterogeneity with evidence pointing to several loci [3].

Oculocutaneous albinism (OCA) is a heterogeneous and autosomal recessive disorder. Based on occurrence of mutation, OCA is identified as nonsyndromic OCA genes (TYR, OCA2, TYRP1, and SLC45A2) and syndromic OCA genes (HPS1, AP3B1, HPS3, HPS4, HPS5, HPS6, DTNBP1, BLOC1S3, PLDN, LYST, MYO5A, RAB27A, and MLPH) [4, 5]. The genes TYR, OCA2, TYRP1, and SLC45A2 are mainly responsible for OCA. Apart from these four genes, other genes are also involved to cause OCA. Several genes encoding melanosomal proteins including SILV, RAB7, TYRP2, SLC24A5, and RAB38 have been considered as good candidates for OCA. However, until recently, no pathological mutations of these genes are reported in human OCA patients [6–9]. Very recently, two new OCA genes are found. Mutations of C10orf11 gene are identified in a family from the Faroe Islands and in a Lithuanian patient [10]. Mutations of SLC24A5 gene are found in a Chinese patient presenting with nonsyndromic OCA [11]. In addition, an OCA locus is mapped to 4q24 in a consanguineous Pakistani family, but the gene has not yet been described [12].

Many polymorphisms have not been experimentally illustrated in terms of their possible disease association. Also, the underlying mechanisms by which a genetic mutation has a deleterious functional effect on its gene product thus causes disease are not yet fully understood. In this study we elucidate the molecular basis of OCA disorder caused by disease-associated mutations. Further we have highlighted a better understanding of the relationships between genetic and phenotypic variation as well as protein structure and function. Our review data further helps in the field of pharmacogenomics to develop a personalized medicine for OCA-associated disorders.

## 2. Prevalence 

Albinism affects one in 20,000 individuals worldwide, but the prevalence of individual subtypes varies among different ethnic backgrounds [13]. OCA-1 is the most common subtype found in Caucasians and accounts for about 50% of cases worldwide [7, 14]. OCA-2, or brown OCA (BOCA), accounts for 30% of cases worldwide and is most common in Africa, where it is estimated to affect one in 10,000 and as many as one in 1,000 in certain populations [15, 16]. This is primarily due to an OCA2 found deletion seen at high frequencies within this population [16–19]. OCA3, or rufous OCA (ROCA), is virtually unseen in Caucasians but affects approximately one in 8,500 individuals from southern Africa or 3% of cases worldwide [14]. OCA-4 is also rare among Caucasians as well as Africans, but worldwide it accounts for 17% of cases and in Japan it is diagnosed in one of four persons affected with OCA [14, 20].

## 3. Symptoms

In general all types of albinism have some lack of pigmentation, but the amount is different depending on the type. OCA1 causes complete absence of pigment in the skin, hair, and eyes, but some individuals may have some degree of pigmentation. OCA1 also affects reduced visual acuity, photophobia (sensitivity to light), and nystagmus (involuntary eye movement). OCA2 causes a minimal to moderate degree of pigmentation in the hair, skin, and eyes. OCA3 has been difficult to identify based on appearance alone. It has been clearly noticeable when a very light-skinned child is born to dark-skinned parents. Ocular albinism affects only the eyes, causing minimal pigmentation. Difficulty controlling eye movements, reduced visual acuity, and nystagmus may occur [21]. OCA6 and OCA7 genes associated with albinism shows the classic visual symptoms and signs but without an obvious change in the pigmentation patterns [10, 11].

## 4. Genes Associated with Oculocutaneous Albinism

### 4.1. TYR

OCA1 (MIM 203100) is caused by mutations in the tyrosinase gene (*TYR*, MIM 606933) on chromosome 11q14.3 [22].* TYR* gene consists of 5 exons spanning about 65 kb of genomic DNA which encodes a protein called tyrosinase and consist of 529 amino acids [23]. TYR (EC 1.14.18.1) is a copper-containing enzyme catalysing the first two steps in the melanin biosynthesis pathway, converting tyrosine to L-dihydroxy-phenylalanine (DOPA) and subsequently to DOPAquinone [24]. Mutations completely abolishing tyrosinase activity result in OCA1A, while mutations rendering some enzyme activity result in OCA1B allowing some accumulation of melanin pigment over time. Almost 303 mutations in TYR are known [25] (Table 1).

Type 1 temperature sensitive oculocutaneous albinism (OCA1-TS) is an extremely rare form of OCA1 characterized by the production of temperature sensitive tyrosinase proteins leading to dark hair on the legs, arms, and chest (cooler body areas) and white hair on the scalp, axilla, and pubic area (warmer body areas). Mutation in temperature sensitive tyrosinase protein is inactivated at 37°C.

### 4.2. OCA2

Mutations in the OCA2 gene (also known as P-gene) (MIM 203200) cause the OCA2 phenotype (MIM 203200) [26]. The OCA2 gene consists of 24 exons (23 coding), spanning almost 345 kb of genomic DNA in the region of 15q11.2-q12. It is highly polymorphic [27] and is suspected to play an important role in human pigmentation [28–30]. This gene encodes P protein, a 110-kDa integral melanosomal protein with 12 predicted transmembrane domains and consists of 838 amino acids [27, 31, 32]. The P protein acts as a precursor to melanin synthesis, within the melanocyte, and serves as a key control point at which ethnic skin colour variation is determined. Moreover, it may stabilize or traffic the melanosomal protein such as tyrosinase which regulate melanosomal pH or serve as a melanosomal tyrosine transporter [26, 33–36]. Currently, in human gene mutation database (HGMD) (http://www.hgmd.org/), 154 mutations in OCA2 gene are listed to cause OCA2 (Table 1). Due to mutation, P protein might disturb the pigmentation characteristics via altering the melanosomal tyrosine or tyrosinase bioavailability or function.

### 4.3. TYRP1

OCA3 is mostly caused by the genetic mutation in* TYRP1 *(MIM 115501) gene. OCA3 is also known as Rufous oculocutaneous albinism. The human* TYRP1* gene consists of 8 exons and 7 introns, spanning almost 15–18 kb of genomic DNA in the region of 9p23 [37–40]. This gene that encodes a protein called tyrosinase-related protein 1 (Tyrp1) has a molecular weight of ~75 kDa and appears to be the most abundant melanosomal protein of the melanocyte [41, 42]. Tyrp1 contains of 537 amino acid residues and shares 40–52% of amino acid homology to tyrosinase protein. Tyrp1 is involved in the maintenance of melanosome structure and affects melanocyte proliferation and cell death [43–46]. Tyrp1 shows tyrosine hydroxylase activity, albeit under low substrate (L-tyrosine) concentration, but no DOPA oxidase activity [47, 48]. Human Tyrp1 also is involved in conversion of L-tyrosine to DOPA with low turnover rates, by the generation of low amounts of DOPA. It is an essential cofactor for tyrosinase activity [49]. Tyrp1 has also been attributed with various other catalytic functions including dopachrome tautomerase (Dct), dihydroxyindole (DHI) oxidase [50], and 5,6-dihydroxyindole-2-carboxylic acid (DHICA) [51]. To date, 16 mutations were found in TYRP1 gene which is responsible for OCA3 (Table 1).

### 4.4. SLC45A2

Mutations in the membrane-associated transporter protein gene (MATP, also known as SLC45A2 and MIM 606202) cause OCA4 (MIM 606574) [52]. MATP consists of 7 exons spanning approximately 40 kb of genomic DNA, mapping to chromosomal position 5p13.3. The SLC45A2/MATP protein consists of 530 amino acids which contains 12 putative transmembrane domains and shows sequence and structural similarity to plant sucrose transporters. It is expressed in melanosomal cell lines [53, 54]. The function of SLC45A2 is still unknown, but studies from Medaka fish show that the SLC45A2 protein plays an important role in pigmentation and probably functions as a membrane transporter in melanosomes [53]. Mutations in SLC45A2 were found for the first time in a Turkish OCA patient [52] and have since been found in German, Japanese, and Korean OCA patients [20, 55–57]. Mutations in SLC45A2 cause misrouting of tyrosinase similar to the cellular phenotype of OCA-2 [58, 59]. To date, 78 mutations were predicted in SLC45A2 gene which is responsible for OCA4 (Table 1).

### 4.5. SLC24A5 and C10orf11

Mutations in* SLC24A5* encode another solute carrier protein. It is a well-known gene in the pigment cell arena and is associated with a new form of OCA, named as OCA6 (MIM 609802).* SLC24A5* gene was located in the chromosomal position of 15q21.1 [11].* SLC24A5* mutations were detected in patients of diverse ethnic origins, thus indicating that OCA6 is not restricted to the Chinese population. It is evident that the cutaneous phenotype was heterogeneous with hair colour changing from white to blond and dark brown [60]. A SNP in* SLC24A5 *(rs1426654) encoding an alanine or threonine at position 111 was detected. Prominently, Thr111 is present in almost all individuals of European American origin, while Ala111 is present in African Asian populations. Thr111 is associated with lighter pigmented skin, thus suggesting an important role of this SNP in the establishment of human pigmentation [61]. Recent results indicate a role of SLC24A5 in the maturation of melanosomes. The assembly of SLC24A5 into melanosomes showed an important role for the melanosomal architecture and to ensure that melanin is synthesized properly. Hence, the lack of SLC24A5 may impair or disrupt melanosomal maturation and melanin biosynthesis [11].

Mutations in the* C10orf11* gene were associated with new form of OCA, designated as OCA7 (MIM 615179).* C10orf11 *was located in the chromosomal position of 10q22.2-q22.3 [10].* C10orf11 *encodes a 198 amino acid protein containing three leucine-rich repeats (LRRs) and one LRR C-terminal (LRRCT) domain. The family of LRRs-containing proteins encompasses members with a variety of functions, including cell adhesion and signalling, extracellular-matrix assembly, neuronal development, and RNA processing [62]. The updated mutation list of these genes was shown in Table 1.

## 5. Other Partial Albinism Disorders and Their Genes

Ocular albinism (OA1) (MIM: 300500) is a form of albinism that affects only the eyes. This disease is caused by mutation in OA1/GPR143 gene which is located on the X chromosome [63] and mutations lists were shown in Table 1. Pigment of the skin and hair is normal or only slightly diluted. Human eyes are severely affected with reduced visual acuity and photophobia. Strabismus or nystagmus is frequently affected with OA1. The fundus and irides are depigmented.

Hermansky-Pudlak syndrome (HPS) (MIM: 203300) is a rare autosomal recessive [64] disorder which results in oculocutaneous albinism (decreased pigmentation), bleeding problems due to storage of an abnormal fat-protein compound (lysosomal accumulation of ceroid lipofuscin), and platelet abnormality (platelet storage pool defect). HPS type 1–9 is caused by mutations in following genes: HPS1, AP3B1, HPS3, HPS4, HPS5, HPS6, DTNBP1, BLOC1S3, and BLOC1S6, respectively, and their updated mutations lists were shown in Table 1. The disease can affect the dysfunctions of lungs, intestine, kidneys, or heart. The major severe form of disorder is pulmonary fibrosis, which routinely exhibits in patient's ages of 40–50 years [65]. The disorder is more common in Puerto Rico [66], where it affects approximately 1 in 1,800 individuals [67].

Chédiak-Higashi syndrome (CHS1) (MIM: 214500) is a rare autosomal recessive disorder caused by the mutation of a lysosomal trafficking regulator protein and leads to a decrease in phagocytosis [68]. The decrease in phagocytosis results in recurrent pyogenic infections, peripheral neuropathy, and partial albinism. The eye, skin, and hair pigment is reduced or diluted in CHS [69, 70]. Mutations in the CHS1 gene (also known as LYST) have been found to be connected with Chédiak-Higashi syndrome (Table 1). CHS1 gene provides instructions for making a protein known as the lysosomal trafficking regulator. Scientists believe that this protein plays a major role in the transport (trafficking) of materials into structures called lysosomes. Lysosomes act as recycling centers within cells. Digestive enzymes are used to break down toxic substances, digest bacteria that invade the cell, and recycle worn-out cell components.

## 6. Computational Screening of Pathological Mutation and Their Molecular Mechanism

**Figure 1:**
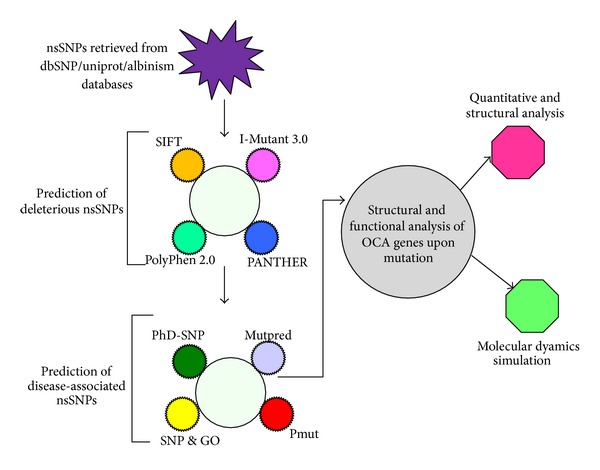
Flow chart of OCA 1–4 gene analysis.

Due to the presence of huge amount of variations data, experimental study of each variant cannot be achieved in a reasonable timescale. Therefore, predictive analysis of the effects of polymorphisms on gene function is needed in order to prioritize the cases that require further study, to elucidate the molecular basis of albinism disorders caused by nonsynonymous mutations. Further, it helps to observe the structural and functional changes of protein upon mutation at atomic level. Screening of nsSNPs of OCA 1–4 genes were analyzed by following computational methods SIFT (deleterious = ≥0.05; tolerated = ≤0.05), PolyPhen (damaging = ≥1.5; benign = ≤1.5), PolyPhen 2.0 (damaging = ≤0.5; benign = ≥0.51), I-Mutant 2.0 (decrease stability = (DDG < 0; increase stability = DDG > 0), I-Mutant 3.0 (decrease = ≤0.5 kcal/moL; Increase = ≥0.5 kcal/moL), and PANTHER (deleterious (>−3); tolerated <−3)). The nsSNPs which were commonly predicted by the above servers were further applied in PHD-SNP, SNP&GO, Pmut, and Mutpred servers to screen the most deleterious and disease-associated mutations in OCA 1–4 genes. The strategy of our investigation is depicted in Figure 1.

The predicted disease-associated mutations could be endorsed with the observed experimental data [71, 72]. The SNP information of OCA 1–4 genes was retrieved from dbSNP/Swiss-prot/albinism database. To understand the atomic arrangement in 3D space, the native and mutant structures of OCA (Tyr, P, Tyrp1, and SLC45A2) proteins were modelled. Molecular dynamics simulation approach was applied to observe the structural and functional behaviour of OCA proteins upon mutation at atomic level. In this review we highlighted the structural and functional consequence of* TYR, OCA2,* and* TYRP1* genes, respectively.

### 6.1. OCA1A

In this study, we rationalized the structural and functional behaviour of tyrosinase protein upon mutation. Based on our investigations we reported the potential candidate SNPs for advanced studies. Out of 57 nsSNPs, four nsSNPs T373K, N371Y, M370T, and P313R in tyrosinase protein were predicted to be functionally significant from our datasets (Table 2).

**Figure 2:**
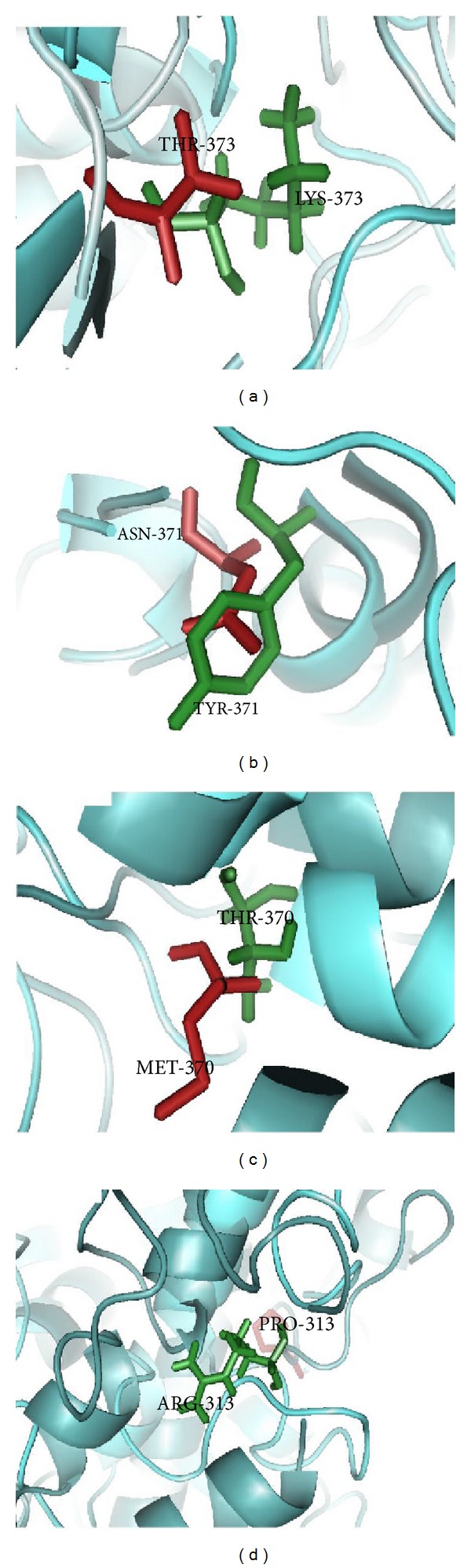
The superimposed structure of native and mutant TYR proteins and mutant residues in stick model: (a) T373K, (b) N371Y, (c) M370T, and (d) P313R.

Threading based approach was applied to predict the model structure of native and mutant TYR proteins. Molecular dynamics simulation approach was applied for refinement of predicted structure of native and mutant TYR proteins. Quantitative and structural approach was performed to rationalized deleterious mutation in TYR gene and molecular mechanism in OCA1A. Due to mutation, TYR structural orientation was altered and became more rigid in nature. This structural disturbance might affect the function of protein and thus the reason to cause OCA1A [73]. The superimposed structure of native and mutant tyrosinase protein and its mutation was displayed in Figures 2(a)–2(d).

### 6.2. OCA2

**Figure 3:**
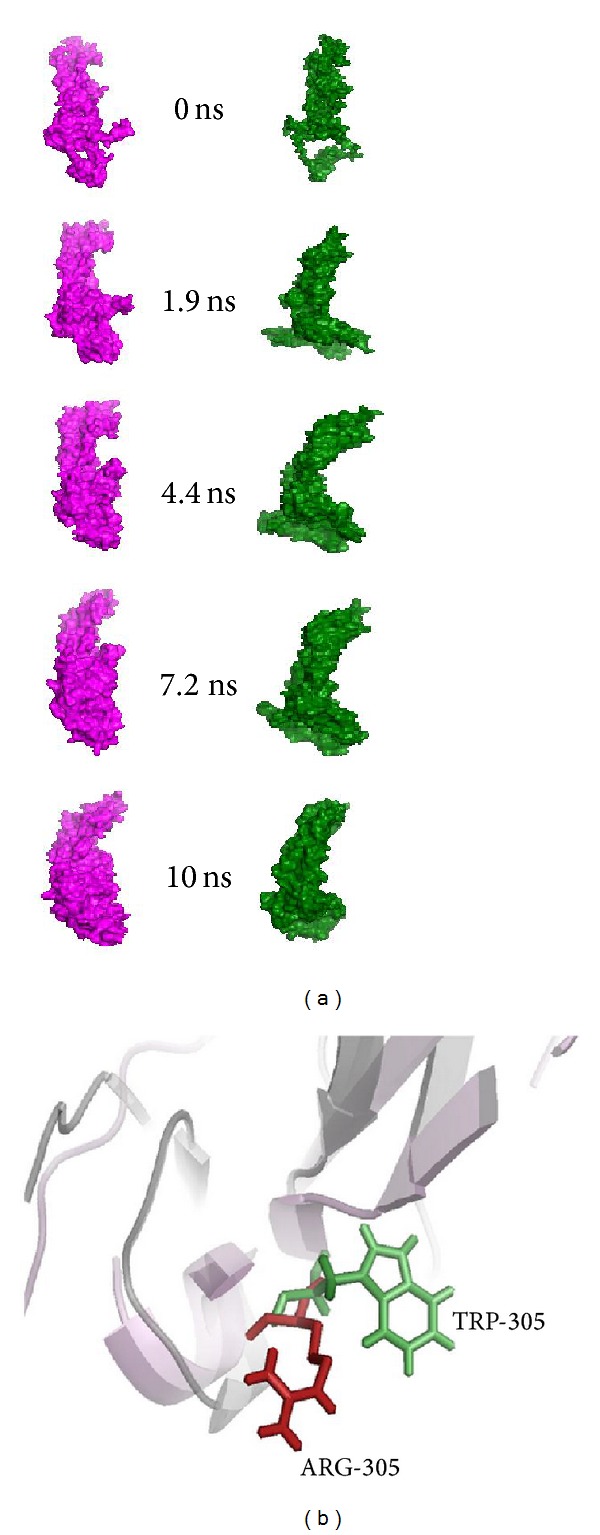
(a) Snapshots of native and mutant P protein conformation at different simulation time steps [74]. (b) The superimposed structure of native and mutant (R305W) P proteins and mutant residues in a stick model.

Using computational approach we screened the most deleterious and disease-associated mutation (R305W) on OCA2 gene from 95 input variants datasets (Table 2). In this analysis, we applied molecular modelling and molecular dynamics simulation approach to examine the structural and functional behaviour of P protein upon mutation. From the molecular dynamics simulation analysis, we confirmed that due to occurrence of mutation the structure loss stability and became more flexible in conformation [74]. Due to flexibility, P protein losses the catalytic function in melanin biosynthesis and might also play a significant role in inducing OCA2 [74]. The superimposed structure of native and mutant P protein and its mutation was shown in Figure 3(b).

### 6.3. OCA3

**Figure 4:**
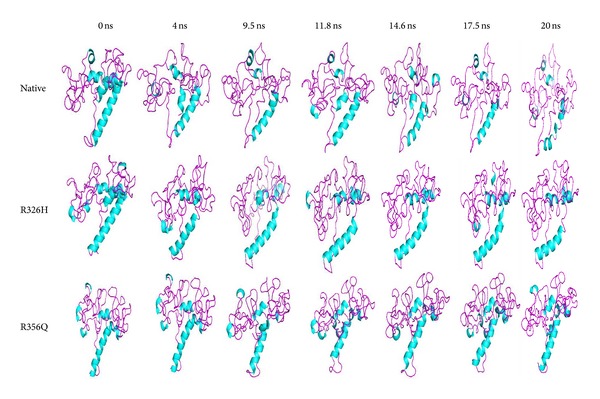
Snapshots of native and mutant (R326H and R356Q) TYRP1 protein conformation at different simulation time steps [75].

In this analysis, we applied* in silico* approach to screen the most disease-associated mutation on TYRP1 gene. Out of 63 nsSNPs, we screened the most disease-associated (R326H and R356Q) mutations on TYRP1 gene (Table 2). The structural analyses of native and mutant Tyrp1 proteins were scrutinized by molecular modelling and molecular dynamics simulation (MDS) approach. From this analysis we confirmed that, TYRP1 protein alter the structural conformation and loss the flexibility behaviour upon mutation [75]. The structural alteration of TYRP1 protein upon mutation was clearly discussed and shown in our previous studies (Figure 4) [75].

**Figure 5:**
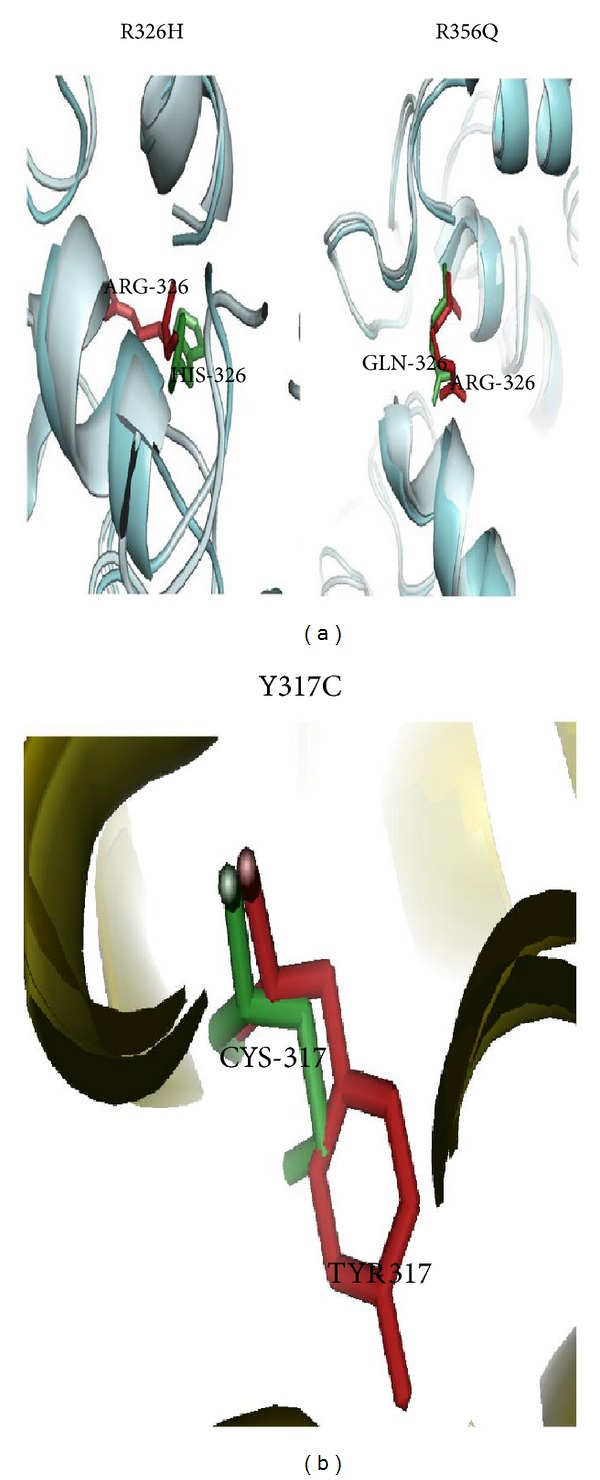
(a) The superimposed structure of native and mutant structures (R326H and R356Q) TYRP1 proteins and mutant residues in a stick model. (b) The superimposed structure of native and mutant (Y317C) SLC45A2 proteins and mutant residue as a stick model.

In Figure 4, both mutant (R326H and R356Q) structures showed an increase in helical content which leads to became more rigid on conformation. Due to rigidity, Tyrp1 protein may lose stability of the tyrosinase protein and modulate its catalytic activity in eumelanin synthesis. It may also disturb the maintenance of melanosome structure and may lead to affect melanocyte proliferation and cell death [75]. The superimposed structure of native and mutant Tyrp1 protein and its mutation residues was shown in Figure 5(a).

### 6.4. OCA4

In this investigation, we implemented multiple computational approaches to identity the most disease-associated mutations in SLC45A2 gene. Based on SIFT, PolyPhen 2.0, I-Mutant 3.0, PANTHER, SNP&GO, PhD-SNP, Pmut, and MutPred servers, we screened the most disease-associated mutation (Y317C) on SLC45A2 gene (Table 2). It may lead to affect the structural conformation and function of the SLC45A2/MATP protein upon mutation. The superimposed structure of native and mutant (Y317C) SLC45A2 protein and its mutation was shown in Figure 5(b). The predicted mutations on TYR, OCA2, and TYRP1 genes and their molecular mechanism can further help wet lab scientist to develop a potent drug target for OCA type 1–3.

## 7. Preclinical Attempts for Albinisms

To date, three drugs were found for albinism related disorders. L-DOPA [76–79] and Adeno associated viral vectors (AAV) [80, 81] helps to treat the OCA1 and OA1. A third therapeutic approved drug, nitisinone, originally devised to treat patients with hereditary tyrosinemia type 1 [82]. This drug has a useful side effect, because it triggers the anomalous accumulation of tyrosine in the blood, thus increasing the concentration of the reagent used by the enzyme tyrosinase (TYR) and resulting in an increased oxidation and improved pigmentation in eyes and skin of mouse models of OCA1B, with some residual tyrosinase activity. Also, the group of Fukai (Osaka, Japan) has suggested the use of aminoglycosides, known to allow the read-through effect over certain nonsense mutations, as a potential therapeutic intervention for some mutations commonly found in albinism [83]. These very promising results soon will be also investigated in humans, in tentative clinical trials. Some people are working to develop a potent drug targets for skin (reduced pigmentation) disorders [84–87]. Additional studies and validations are still required before transferring any of these therapeutic proposals, here listed, to routine treatment for human subjects.

## 8. Concluding Remarks

Albinisms are studied for many years as a congenital rare disease without cure, where a person born with albinism will ultimately die with the same disease. This situation has now changed and will continue improving in the near future. Oculocutaneous albinism is caused by all the ethnic backgrounds of humans. It is caused by the mutation in OCA genes. Due to mutation, the melanin biosynthesis pathway inhibits the action of melanin synthesis, which leads to albinism. Our predictions suggest a significant computational approach to detect the OCA 1–3 (TYR, TYRP1, OCA2, and SLC45A2 genes) associated SNPs from the large SNP dataset and reduce the expenses in experimental depiction of pathological mutations. Most of this pathological evidence identified in OCA has been reported and still we lack in understanding their associated molecular mechanism. To develop an efficient therapeutic approach, it is highly necessary to understand the detailed knowledge of their molecular mechanisms, to illustrate their structural and functional behaviours in detail. The SLC45A2, SLC24A2, and C10orf11 genes structural and functional behaviours are still unknown. We must also continue to elucidate the high resolution 3D structures and their localization patterns of OCA genes, as these data will aid in developing a potent drug target for albinism related disorders.

## Figures and Tables

**Table 1:** Mutations detected for albinism associated genes.

Albinism	Gene	Chromosome location	Mutations
OCA1	TYR	11q14-q21	303
OCA2	OCA2	15q11.2-q12	154
OCA3	TYRP1	9p23	16
OCA4	SLC45A2	5p13.3	78
OCA5	ND	4q24	1
OCA6	SLC24A5	15q21.1	2
OCA7	C10ORF11	10q22.2-q22.3	1
OA1	GPR143	Xp22.3	114
LYST	CHS1	1q42.1-q42.2	53
HPS1	HPS1	10q23.1-q23.3	31
AP3B1	HPS2	5q14.1	20
HPS3	HPS3	3q24	7
HPS4	HPS4	22cen-q12.3	13
HPS5	HPS5	11p14	11
HPS6	HPS6	10q24.32	9
HPS7	DTNBP1	6p22.3	2
HPS8	BLOC1S3	19q13.32	2
HPS9	BLOC1S6	15q21.1	1

Source: human gene mutation database, 05 Dec, 2013; ND: not determined.

**Table 2:** Gene names, chromosome location, protein name, and predicted mutations of OCA1–4.

Disease types	Gene	Chromosome location	Protein	Predicted mutations
OCA1A	TYR	11q14.3	Tyrosinase	T373K, N371Y, M370T, P313R
OCA2	OCA2	15q11.2-q12	P protein/melanocyte-specific transporter protein	R305W
OCA3	TYRP1	9p23	Tyrosinase related protein-1	R326H, R356Q
OCA4	SLC45A2/MATP	5p13.3	Membrane-associated transporter protein	Y317C
